# The Effect of Flow-Induced Vibration on Heat and Mass Transfer Performance of Hollow Fiber Membranes in the Humidification/Dehumidification Process

**DOI:** 10.3390/membranes11120918

**Published:** 2021-11-24

**Authors:** Zhenxing Li, Bo Chen, Caihang Liang, Nanfeng Li, Yunyun Zhao, Chuanshuai Dong

**Affiliations:** 1 School of Mechanical and Electrical Engineering, Guilin University of Electronic Technology, Guilin 541004, China; lzx@guet.edu.cn (Z.L.); 1801302004@mail.guet.edu.cn (B.C.); linanfengx@gmail.com (N.L.); 2 Guangdong Province Key Laboratory of Distributed Energy System, Guangdong Provincial Engineering Research Center of Distributed Energy Systems, Dongguan University of Technology, Dongguan 523808, China; 3 School of Materials Science and Engineering, Guilin University of Electronic Technology, Guilin 541004, China; yunyzhao@163.com; 4 Key Laboratory of Enhanced Heat Transfer and Energy Conservation of Education Ministry, School of Chemistry and Chemical Engineering, South China University of Technology, Guangzhou 510641, China; dongcs@scut.edu.cn

**Keywords:** flow-induced vibration, pulsating flow, fiber deformation, two-way fluid solid interaction, heat and mass transfer

## Abstract

Cross-flow hollow fiber membranes are commonly applied in humidification/dehumidification. Hollow fiber membranes vibrate and deform under the impinging force of incoming air and the gravity of liquid in the inner tube. In this study, fiber deformation was caused by the pulsating flow of air. With varied pulsating amplitudes and frequencies, single-fiber deformation was investigated numerically using the fluid–structure interaction technique and verified with experimental data testing with a laser vibrometer. Then, the effect of pulsating amplitude and frequency on heat and mass transfer performance of the hollow fiber membrane was analyzed. The maximum fiber deformation along the airflow direction was far larger than that perpendicular to the flow direction. Compared with the case where the fiber did not vibrate, increasing the pulsation amplitude could strengthen *Nu* by 14–87%. Flow-induced fiber vibration could raise the heat transfer enhancement index from 13.8% to 80%. The pulsating frequency could also enhance the heat transfer of hollow fiber membranes due to the continuously weakened thermal boundary layer. With the increase in pulsating amplitude or frequency, the *Sh* number or *E*_m_ under vibrating conditions can reach about twice its value under non-vibrating conditions.

## 1. Introduction

A hollow fiber membrane is a membrane formed by the intersection of functional fiber materials and separation membrane technology. It has the advantages of high packing density, small occupying space, simple membrane module structure, and relatively low cost. Hollow fiber membrane technology has become key in the fields of environmental protection, resource recovery, the new energy industry, and the upgrading of traditional industries. In recent years, hollow fiber membrane modules have become widely used in wastewater treatment [1,2,3], membrane extraction [4,5,6,7], gas separation [8,9,10], and air humidification [11,12,13,14,15] and dehumidification processes [16,17,18,19,20].

Generally, a hollow fiber membrane is a semi-permeable unit with high permeability and selectivity of target compounds [5]. The structure of a hollow fiber membrane module is similar to that of a shell and tube heat exchanger, where the hollow fibers act as the tube bundle. In the humidification/dehumidification process, liquid usually flows into the hollow fibers and air flows into the shell side. The water vapor can permeate through the membrane, but liquid water will be intercepted by the membrane. Thus, the calcium and magnesium ions in water, which are harmful to human health, will not enter the air stream. The heat and mass transfer characteristics of the hollow fiber membrane bundle for humidification/dehumidification have been investigated by many researchers theoretically [18,21,22,23,24] and experimentally [25,26,27,28].

When a hollow fiber membrane is used in water treatment, gas separation, and other fields, the hollow fibers are impinged by the fluid in the shell side. Then, fibers deform and vibrate due to the impinging of the incoming fluid. Fiber deformation will conversely affect the flow field and heat mass transfer performance during operation. Therefore, how the flow-induced vibration affects the stress distribution and heat and mass transfer performance of hollow fiber membranes is worthy of study. The effect of heat transfer enhancement caused by flow-induced vibration has been observed in elastic tube bundles, and the heat transfer coefficient can be enhanced by about 20–30% compared with steady flow conditions [29,30,31,32]. Unlike the rigid metallic tubes in shell and tube heat exchangers, hollow fiber membranes are flexible tube bundles that are prone to vibration due to the influence of fluid flow and gravity. During the humidification/dehumidification process, the airflow causes fiber vibration and deformation. The fiber vibration then affects the flow field, thus influencing the heat and mass transfer performance of the fiber bundle. This is a typical fluid–solid coupling problem.

The effect of vibrations on submerged hollow fiber membranes has been studied experimentally with the aim of mitigating membrane fouling [33,34,35,36,37,38,39,40,41]. These studies mainly focused on the influence of membrane vibration on the critical flux, transmembrane pressure, and other parameters that reflect the degree of membrane fouling but not the influence of fluid–structure coupling on the heat and mass transfer performance of hollow fiber membranes. Zamani et al. [42] proposed an analytical model to predict the wall shear rate at the membrane surface caused by longitudinal vibration. Liu et al. [43] investigated aeration-induced fiber movement on surface shear stress and flux in submerged hollow fiber membrane systems using the fluid–structure interaction (FSI) approach. Huang et al. [44] conducted a numerical study of the fluid flow and heat transfer characteristics of curved hollow fiber membrane bundles for air humidification/dehumidification. The hollow fibers were curved at certain deformed heights and angles in their model. However, the deformed heights and angles of the fiber were assumed according to the empirical values obtained in their study.

The fluid flow, stress, and heat and mass transfer in hollow fiber membrane bundles under flow-induced vibration have not been fully investigated. Fiber vibration frequently changes the stress acting on the fiber. Therefore, under these conditions, the fiber will be abraded at both ends and its lifetime will be shortened. From a mechanical point of view, reducing the effective length of the fiber can reduce the stress at both ends of the fiber and prolong its lifetime. Stress distribution on the fiber is important for the membrane’s lifetime, and the Nusselt number (*Nu*) and Sherwood number (*Sh*) across the fiber are key parameters in the design of hollow fiber membrane modules. In this study, the effects of fiber vibration on stress, *Nu*, and *Sh* for flow across a hollow fiber were investigated using the FSI approach. In this way, the influence of the pulsating amplitude and frequency of the incoming air on *Nu* and *Sh* was studied.

## 2. Methodology

### 2.1. Physical Model

**Figure 1:**
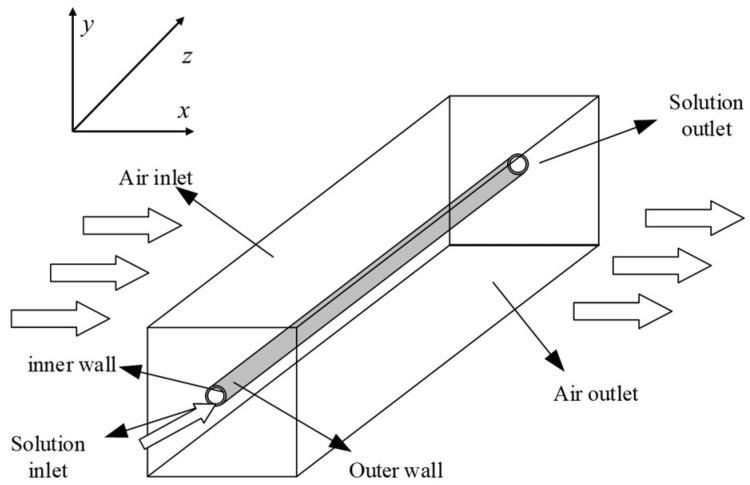
The physical model of fluid–solid coupling for single-fiber vibration.

A physical model of single-fiber vibration is shown in Figure 1. The computational domain is divided into three zones: the airflow zone outside the fiber, the liquid flow zone inside the fiber, and the solid zone that represents the hollow fiber membrane. Under practical conditions, air flows into the computational domain with a pulsating speed and impinges on the fiber. Then, the fiber begins to vibrate under the force of the air, self-gravity, and gravity from the inner fluid. Continuous vibration of the fiber will affect the flow field of the air. This is a classic fluid–structure coupling phenomenon that involves both fluid dynamics and structural mechanics. When addressing this phenomenon, the computational fluid dynamics (CFD) method treats the fiber as a rigid tube that will not deform; thus, the transient impact of fiber deformation on the flow field cannot be solved. In this study, the FSI approach was applied to assess the interaction between the fiber and the fluid. In the FSI approach, the flow field and the force acting on the fiber are calculated by the CFD method. Then, the fiber deformation is calculated by transient structural analysis, which, in turn, affects the CFD calculation of the flow field. The effect of vibration on heat and mass transfer in the inner tube was ignored in this study. Detailed dimensions of the model are listed in Table 1.

### 2.2. Governing Equations

To establish a suitable mathematical model for the simulation, the following assumptions were made: (1) the fluid is an incompressible Newtonian fluid; (2) the physical parameters are constant; (3) the heat effect caused by viscous dissipation during fluid flow is ignored.

#### 2.2.1. Governing Equation for the Solid Domain

The domain of the hollow fiber membrane is solid. The response of the hollow fiber membrane to the internal and external loads can be defined by partial differential equations. The momentum equations of the solid domain in the *x*, *y*, and *z* directions are depicted as:

(1) $$\frac{\partial \sigma_{xx}}{\partial x}+\frac{\partial \sigma_{xy}}{\partial y}+\frac{\partial \sigma_{xz}}{\partial z}+b_{x}=\rho_{s}\frac{\partial^{2}x}{\partial t^{2}}$$

(2) $$\frac{\partial \sigma_{yx}}{\partial x}+\frac{\partial \sigma_{yy}}{\partial y}+\frac{\partial \sigma_{yz}}{\partial z}+b_{z}=\rho_{s}\frac{\partial^{2}y}{\partial t^{2}}$$

(3) $$\frac{\partial \sigma_{zx}}{\partial x}+\frac{\partial \sigma_{zy}}{\partial y}+\frac{\partial \sigma_{zz}}{\partial z}+b_{z}=\rho_{s}\frac{\partial^{2}z}{\partial t^{2}}$$

where *σ* represents the stress (Pa), *ρ*_s_ is the density of the hollow fiber membrane (kg/m^3^), *b* is the acceleration of the volume element (m^2^/s), and *t* is time (s).

The energy conservation equation is as follows:

(4) $$\frac{\partial e}{\partial t}=\frac{1}{\rho }(\sigma_{xx}\frac{\partial \varepsilon_{xx}}{\partial x}+\sigma_{yy}\frac{\partial \varepsilon_{yy}}{\partial y}+\sigma_{zz}\frac{\partial \varepsilon_{zz}}{\partial z}+2\sigma_{xy}\frac{\partial \varepsilon_{xy}}{\partial x}+2\sigma_{yz}\frac{\partial \varepsilon_{yz}}{\partial y}+2\sigma_{zx}\frac{\partial \varepsilon_{zx}}{\partial z})$$

where *e* is the energy (J) and *ε* is the dissipation rate of the turbulent kinetic energy (m^2^/s^3^).

#### 2.2.2. Governing Equation for the Fluid Domain

In the fluid domain, there are two kinds of fluids: air and solution. Both air and solution are defined as Newtonian fluids, which are incompressible, and their thermophysical properties are assumed to be unchanged. The RNG k-epsilon model was chosen to describe the flow state, which can be found in [44]. The governing equation for the fluid is as follows:

(5) $$\frac{\partial }{\partial t}\left(\rho_{a}\vec{V_{a}}\right)+\nabla \cdot \left(\rho_{a}\vec{V_{a}}\vec{V_{a}}\right)=-\nabla p+\nabla \cdot \left(\sigma \right)+\rho_{f}\vec{g}+\vec{F}$$

where *p* is the static pressure (Pa), *ρ*_*f*_*g* is gravity (N), and *F* is the external volume force (N).

Dynamic grid technology theory was applied to capture the deformation of the solid domain based on the Lagrangian–Euler method. Therefore, the fluid–solid coupling problem can be solved through the deformed grid using the dynamic grid function. The control equations for momentum, energy, and species transport are [45]:

(6) $$\nabla (V_{a}-V_{am})=0$$

(7) $$\frac{\partial V_{a}}{\partial t}+\left(V_{a}-V_{am}\right)\nabla V_{f}+\frac{\nabla p}{\rho }=v\nabla^{2}V_{a}$$

(8) $$\rho_{a}C_{p,a}\frac{\partial T_{a}}{\partial t}+\rho_{a}C_{p,a}(V_{a}-V_{am})\nabla T_{a}=\lambda_{a}\nabla^{2}T_{a}$$

(9) $$\rho_{a}\frac{\partial \omega_{a}}{\partial t}+\nabla \cdot \left[\rho_{a}(u_{a}-u_{am})\omega_{a}\right]=-\nabla \cdot J_{a}$$

where *V*_*am*_ is the velocity of the air domain grid (m/s), *V*_*a*_ − *V*_*am*_ is the velocity of the air relative to the grid (m/s), *C*_*p*,*a*_ is the specific heat capacity of the air at constant pressure (J/kg∙K^−1^), *λ*_*a*_ is the thermal conductivity of air (W/m∙K^−1^), and *ω*_*a*_ is the mass fraction of water vapor in humid air. The entire diffusion process is driven by the difference in concentration and temperature. According to Fick’s theory, ***J***_*a*_ is defined as:

(10) $$J_{a}=-\rho_{a}D_{v,a}\nabla \omega_{a}-D_{v,T}\frac{\nabla T}{T}$$

where *D*_*v*,*a*_ is the binary diffusion coefficient of water vapor in the air (m^2^/s) and *D*_*v*,*T*_ is the thermal diffusion coefficient of water vapor driven by the temperature field (m^2^/s).

### 2.3. Boundary Conditions

When performing fluid–solid coupling calculations, the velocity, displacement, and stress of the fluid–solid interface need to meet certain conditions to balance the forces on both sides. The boundary conditions of the interface are:

(11) $$\left\{ \begin{array}{l} d_{s}=d_{f}\\ u_{s}=u_{{}_{f}}\\ n\cdot \sigma_{s}=n\cdot \sigma_{f} \end{array}\right.$$

where *d* is the displacement (m) and *n* refers to the unit normal vector of stress, which is perpendicular to the interface of fluid and solid.

Fiber tubes produce a vibration response under the effect of pulsating flow. Due to the existence of damping, there will be energy loss at the fluid–solid interface. The dynamic response equation of the system is:

(12) $$M_{f}\ddot{p}+C_{f}\dot{p}+K_{f}p+\rho R^{T}\ddot{d}=0$$

Pulsating air causes the hollow fiber membrane (the solid domain) to vibrate, and the force equation is:

(13) $$M_{s}\ddot{d}+C_{s}\dot{d}+K_{s}d=\sigma_{f}+\sigma_{s}$$

(14) $$\left[\begin{array}{cc} M_{s} & 0\\ \rho R^{T} & M_{f} \end{array}\right]\left\{\begin{array}{c} \ddot{d}\\ \ddot{p} \end{array}\right\}+\left[\begin{array}{cc} C_{s} & 0\\ 0 & C_{f} \end{array}\right]\left\{\begin{array}{c} \dot{d}\\ \dot{p} \end{array}\right\}+\left[\begin{array}{cc} K_{s} & 0\\ 0 & K_{f} \end{array}\right]\left\{\begin{array}{c} d\\ p \end{array}\right\}=\left\{\begin{array}{c} \sigma_{s}+\sigma_{f}\\ 0 \end{array}\right\}$$

where *M*, *C*, *K*, and *R*^*T*^ are the mass matrix, damping matrix, stiffness matrix and coupling matrix, respectively.

Air flows along the positive direction of the *x*-axis, and the solution flows along the positive direction of the *z*-axis. The velocity of the air is defined as a sine function, and its definition is:

(15) $$V_{a}=Asin(2\pi Bt)+C$$

where *A* is the amplitude, *B* is the frequency, and *C* is the phase.

The main object of this research was air in the shell side, with the flow rate of liquid in the inner tube remaining unchanged. The amplitude and frequency of pulsating flow were altered in this simulation; detailed setups are given in Table 2.

The temperature and humidity in the inlet are defined as:

(16) $$T^{*}=0,\;\omega^{*}=0$$

At the fiber outer surface, the uniform temperature and humidity condition is applied.

(17) $$T^{*}=1,\;\omega^{*}=1$$

At the air outlet, the boundary condition is defined as:

(18) $$\frac{\partial u_{x}}{\partial z}=\frac{\partial u_{y}}{\partial z}=\frac{\partial u_{z}}{\partial z}=0,\;\frac{\partial T^{*}}{\partial z}=0,\;\frac{\partial \omega^{*}}{\partial z}=0$$

The parameter setups and fiber material properties in the calculation are shown in Table 3. The main purpose of our current research was to study the heat and mass transfer performance of a single fiber under flow-induced vibration through the two-way FSI method. The solution method was based on constant wall temperature/concentration boundary conditions; therefore, the results of the dimensionless heat and mass transfer coefficient are suitable for a wide range of applications, such as gas separation, seawater desalination, and sewage treatment.

### 2.4. Solution Strategy

**Figure 2:**
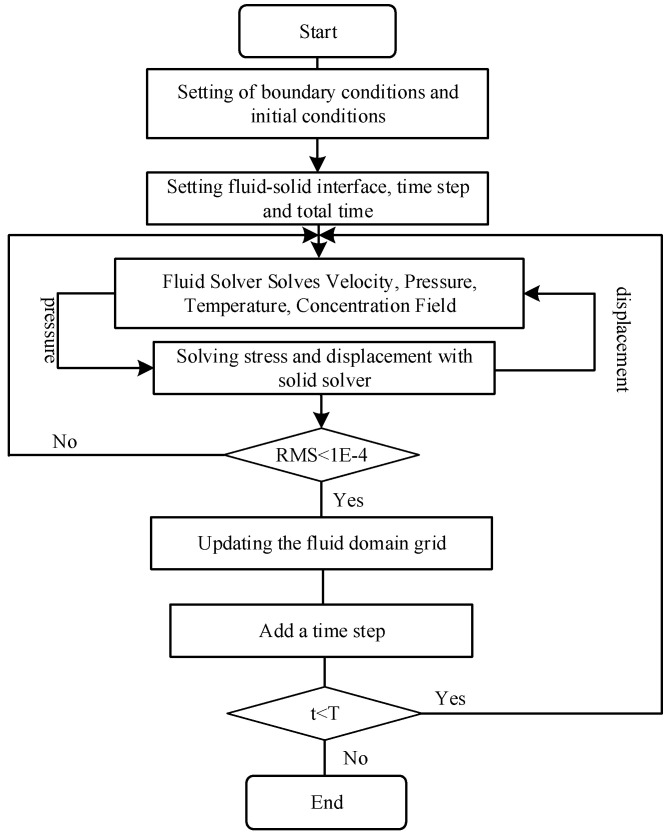
The procedure of the fluid–solid coupling calculation.

Under practical conditions, even at Reynolds numbers ranging from 50 to 350 [44], turbulence will be generated when air flows across the fiber bundle. The entire calculation process is shown in Figure 2.

(1) According to the previously specified boundary conditions, the flow velocity, pressure data, temperature data, and concentration data of the internal and external flow fields were calculated. At the time t = 0, the pressure data are transmitted to the solid structure solver through the system coupling communication module. Additionally, no deformation information on the solid domain is transmitted to the fluid domain.

(2) The transient structure solver receives the pressure data of the fluid domain transmitted by the system-coupling calculation module. Then, the transient structure solver applies pressure to the solid surface as a load and calculates the deformation of the fiber at this time step. The solver judges convergence according to the root mean square (RMS) value in this time step. When RMS value of deformation changes less than 1 × 10^−4^, the result at this time step is judged as converged. The deformation data are transmitted through the system coupling to the fluid domain, updating the dynamic mesh, and changing the shape of the fluid domain.

(3) The system coupling calculation module enters the next time step, repeats the first and second steps in the fluid domain after the mesh is updated, and keeps looping until the pre-specified end time is reached.

(19) $$\nabla \cdot \left(\theta \cdot \nabla u_{fm}\right)=0$$

where *θ* is the grid diffusion coefficient and *u_fm_* is the velocity of the grid movement.

### 2.5. Mesh Independence Check

Two-way fluid–solid coupling calculations require considerable computing resources and time. Therefore, the choice of an appropriate grid number can effectively improve computational efficiency. A mesh independence check was performed in this study, and the calculation data for different grid systems are given in Table 4. In total, five grid systems were generated for calculation. The temperature and mass fraction of water vapor in the air outlet were selected to check the computational accuracy. The results show that when the grid number is larger than 293,025, the deviation for temperature and mass fraction of water vapor is less than 0.01%. Considering the calculation power and time consumption, a grid system with node number 293,025 was selected for calculation.

### 2.6. Performance Criterion

The Reynolds number is:

(20) $$Re=\frac{\rho V_{m}D_{h}}{\mu }$$

where *V*_m_ is the average velocity of incoming air (m/s), and *D*_h_ is the hydraulic diameter (m). For airflow, *D*_h_ is equal to the fiber outer diameter. For solution flow, *D*_h_ is equal to the inner fiber diameter.

The performance criterion for the heat and mass transfer is represented as Nusselt number, Sherwood number, heat transfer enhancement index, and mass transfer enhancement index. The definition of the Nusselt number is:

(21) $$Nu=\frac{hD_{h}}{\lambda }$$

where *h* is the convective heat transfer coefficient (W/m^2^⋅K^−1^). *h* is calculated as:

(22) $$h=\frac{\rho c_{p}V_{i}A_{i}(T_{i}-T_{o})}{A_{\mathrm{mem}}\Delta T_{\log}}$$

where the subscripts *i* and *o* represent the inlet and outlet, respectively, *A_i_* is the area of the inlet, *A*_mem_ is the total area of the hollow fiber membrane (m^2^), and Δ*T*_log_ is the logarithmic mean temperature difference.

(23) $$\Delta T_{\log}=\frac{\left(T_{W}-T_{i})-(T_{W}-T_{o}\right)}{\ln\left[\left(T_{W}-T_{i})/(T_{W}-T_{o}\right)\right]}$$

where the subscript *W* means wall.

The Sherwood number is calculated by:

(24) $$Sh=\frac{k_{a}d}{D_{\mathrm{va}}}$$

where *k*_a_ is the convective mass transfer coefficient (m/s). *k*_a_ is calculated as:

(25) $$k_{a}=\frac{\rho V_{a}\left(\omega_{i}-\omega_{o}\right)}{A_{mem}\Delta \omega }$$

where Δ*ω* is the logarithmic mean humidity difference:

(26) $$\Delta \omega =\frac{\left(\omega_{W}-\omega_{i})-(\omega_{W}-\omega_{o}\right)}{\ln\left[\left(\omega_{W}-\omega_{i})/(\omega_{W}-\omega_{o}\right)\right]}$$

The heat transfer enhancement index is defined as [46]:

(27) $$E_{h}=\frac{\Delta T_{c}-\Delta T_{uc}}{\Delta T_{uc}}$$

(28) $$\Delta T_{c}=T_{c,i}-T_{c,o}$$

(29) $$\Delta T_{uc}=T_{uc,i}-T_{uc,o}$$

where Δ*T*_*c*_ (K) is the temperature difference between air inlet and outlet when the fiber vibrates, and Δ*T_u__c_* (K) is the temperature difference when the fiber does not vibrate.

The mass transfer enhancement index is defined as:

(30) $$E_{m}=\frac{\Delta \omega_{c}-\Delta \omega_{uc}}{\Delta \omega_{c}}$$

(31) $$\Delta \omega_{c}=\omega_{c,i}-\omega_{c,o}$$

(32) $$\Delta \omega_{uc}=\omega_{uc,i}-\omega_{uc,o}$$

where Δ*ω*_*c*_ is the mass fraction difference of water vapor between the air inlet and outlet when the fiber vibrates and Δ*ω_u__c_* is the mass fraction difference of water vapor when the fiber does not vibrate.

## 3. Experiment Verification

**Figure 3:**
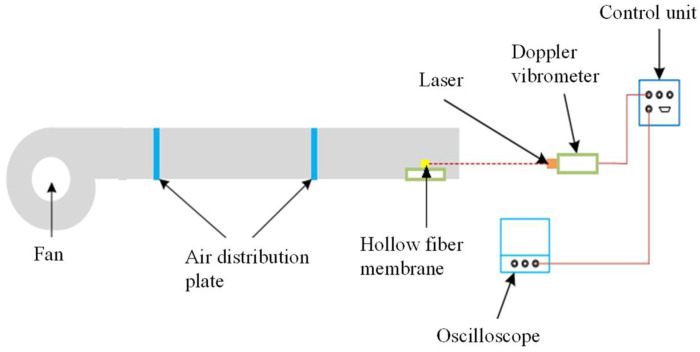
The test section for single-fiber vibration.

The experiment was conducted to verify the fiber vibration calculated by the numerical model. The experimental device for measuring the flow-induced vibration of a single hollow fiber membrane is shown in Figure 3. A hollow fiber membrane made of polyvinylidene fluoride with a nominal pore size of 0.4 μm was used for the test. The inner tube of the fiber was filled with water, and both ends of the fiber penetrated an acrylic plate through a hole and were then fixed with epoxy glue. The hollow fiber membrane had an outer diameter of 1.7 mm, an inner diameter of 1.5 mm, and a length of 10 cm. The air volume flow rate was controlled using a draught fan with variable frequency (SIEMENS^®^ 1LE001, SIEMENS Co. Ltd., Berlin, Germany). To ensure the accuracy of the numerical over a wide *Re* range, the incoming air velocity was tested from 0 to 2.5 m/s. The corresponding Reynolds number was 0–275. To ensure the measurement accuracy of the air velocity, the data were tested after 1 min of adjusting the fan frequency. Additionally, for each Reynolds number, three wind speeds were recorded with a time interval of 30 s. A circular tube with a diameter of 15 cm and a length of 5 m was used as the test flow channel. The incoming air velocity was measured using the Testo-425 hot wire anemometer. The flow-induced fiber deformation was tested using the laser vibrometer (melectro^®^ V-100lm type-DA) produced by DENSHIGIKENQ Co., Ltd. (Osaka, Japan). This laser vibrometer has high precision and good linearity. It can ensure the consistency of amplitude in the high-frequency range. This apparatus uses laser reflection to measure the fiber deformation; therefore, it is not limited by distance and adds no additional mass to the test section, so it is suitable for measuring hollow fiber membranes.

The pressure drop of a single fiber is difficult to measure accurately because it is much lower than the pressure drop of airflow through tube bundles. Additionally, it is hard to test the pressure drop difference between a vibrating and non-vibrating fiber. The arrangement of the fiber will greatly affect the velocity field of flow across a fiber bundle; thus, the pressure drops for flow across a single fiber or fiber bundle are quite different. Obtaining the pressure drop of flow across a tube bundle is more meaningful. Thus, the pressure drop was not tested in this study.

## 4. Results and Discussions

### 4.1. Model Validation

To validate the numerical model, the amplitude of the hollow fiber was tested and compared with the numerical results. The tested air velocity was higher than that used in actual operating conditions. The selected air velocities were 1 m/s, 1.5 m/s, 2 m/s, and 2.5 m/s, corresponding to *Re* numbers of 110, 165, 220, and 275, respectively. For each *Re* number, deformation data of fiber were tested five times. The results for fiber deformation in the *x* and *y* directions are given in Table 5. The maximum deviations between the experimental and numerical data in the *x* direction were −3.70%, 4.17%, −2.68%, and −2.07% for each *Re* number, and in the *y* direction, they were 7.69%, −6.52%, −3.85%, and 3.74%. The numerical data fitted well with the experimental results; therefore, this model is sufficiently accurate to predict the flow-induced vibration of hollow fiber membranes.

### 4.2. The Effect of Air Pulsation Amplitude on Stress Distribution of the Fiber

**Figure 4:**
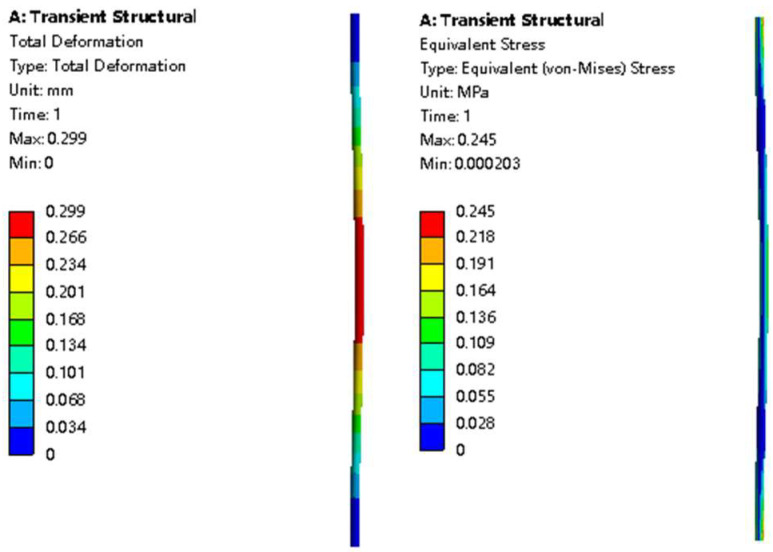
Deformation and stress distribution on the fiber (*Re* = 165).

Fiber vibration causes frequent force variations at the fixed end of the fiber. Therefore, the fiber will be abraded, and its lifetime will be shortened. Thus, we need to know the stress distribution on the fiber to guide membrane module design. The deformation and stress distribution on the fiber are shown in Figure 4. As can be seen from the figures, the maximum total deformation occurred in the center of the fiber. As for the stress distribution, the maximum value was at both ends of the fiber, and the second highest value was at the center of the fiber. This is consistent with our conjecture. The two ends of the fiber were fixed; therefore, the stress here was largest in the vibration process. Additionally, the wind speed at the center of the fiber was the greatest; thus, the stress there was relatively high. Detailed stresses for various *Re* values are given in Table 6. This approach can predict the stress distribution caused by flow-induced fiber vibration and thus provide data to establish the relationship between stress conditions and fiber lifetimes.

### 4.3. The Effect of Air Pulsation Amplitude on Heat Transfer Performance

**Figure 5:**
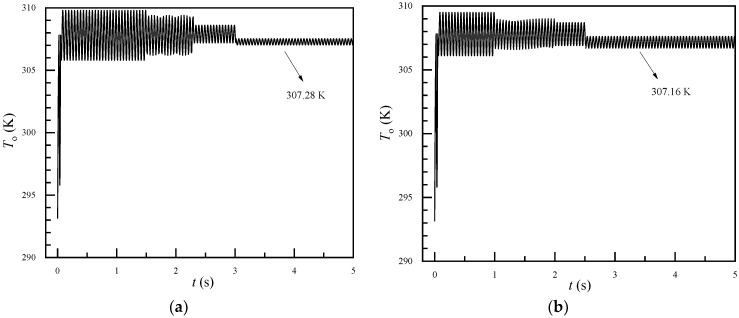
Transient outlet air temperature (*V*m = 1 m/s). (**a**) Ignoring fiber vibration. (**b**) Considering fiber vibration.

**Figure 6:**
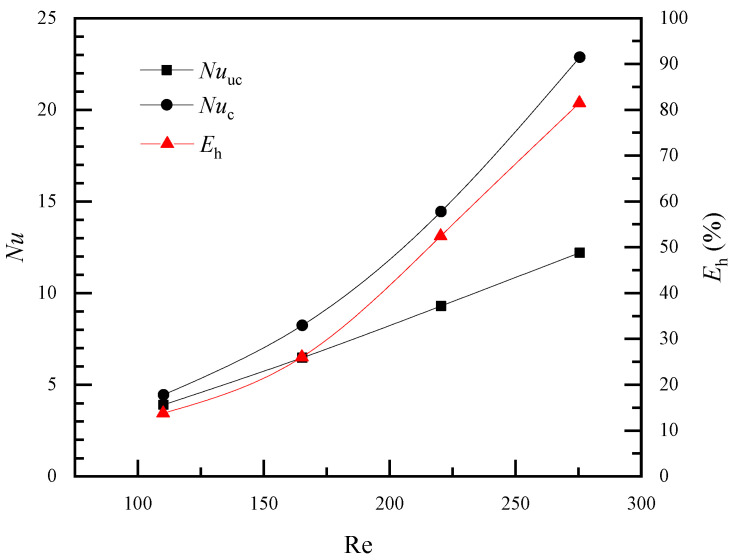
Vibrations of *Nu* and *E_h_* with air pulsation amplitude (*f* = 20 Hz).

The validated numerical model was used to simulate heat transfer in the humidification process. In this simulation, water flows into the fiber tube at a velocity of 0.08 m/s, corresponding to a laminar flow. Additionally, the incoming air velocities were 1 m/s, 1.5 m/s, 2 m/s, and 2.5 m/s, as depicted in Table 2. The air pulsation frequency was set to 20 Hz. Figure 5 shows the transient variation of outlet air temperature with or without fiber vibration (*V*_m_ = 1 m/s, *Re* = 110). For Figure 5a, only the incoming air pulsated in a sinusoidal waveform, and the FSI approach was not used in the calculation; thus, the fiber did not vibrate. In Figure 5b, the fiber was modeled with the FSI approach and vibrated with the inlet air. The outlet air temperature fluctuated with the sinusoidal waveform, similarly to the pulsation of the inlet air velocity. As time progressed, the pulsation amplitude of the air outlet temperature gradually decreased. In the later stage, the air outlet temperature was 307.16K when considering the vibration of the hollow fiber, and 307.28K when the fiber vibration is ignored. Additionally, the heat transfer enhancement index reached 13.8%. Detailed *Nu* and *E_h_* values are given in Figure 6. The maximum heat transfer enhancement index could be as high as 80%.

### 4.4. The Effect of Air Pulsation Frequency on Heat Transfer Performance

**Figure 7:**
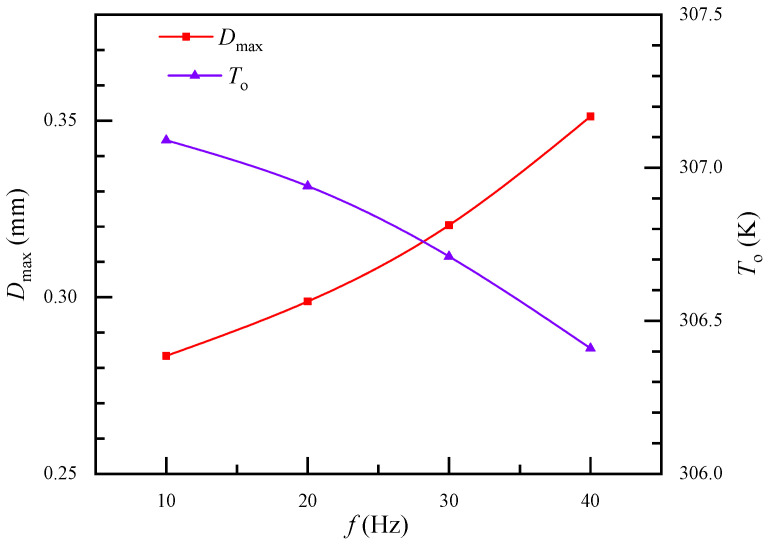
The effect of air pulsating frequency on maximum fiber deformation and air outlet temperature.

The effect of air pulsating frequency on heat transfer performance was also investigated. Parameters A and C in Equation (15) are assumed to be 1 and 1.5 in this section, respectively. Figure 7 shows the air effect of pulsating frequency on maximum fiber deformation and outlet air temperature. The heat transfer enhancement index increased by 65.9% when the pulsating frequency increased from 10 Hz to 40 Hz. The maximum fiber deformation slightly increased with pulsating frequency, but the outlet air temperature apparently declined. This indicates that the pulsating frequency of incoming air had little effect on fiber deformation. Therefore, heat transfer enhancement may be caused by the pulsating frequency.

**Figure 8:**
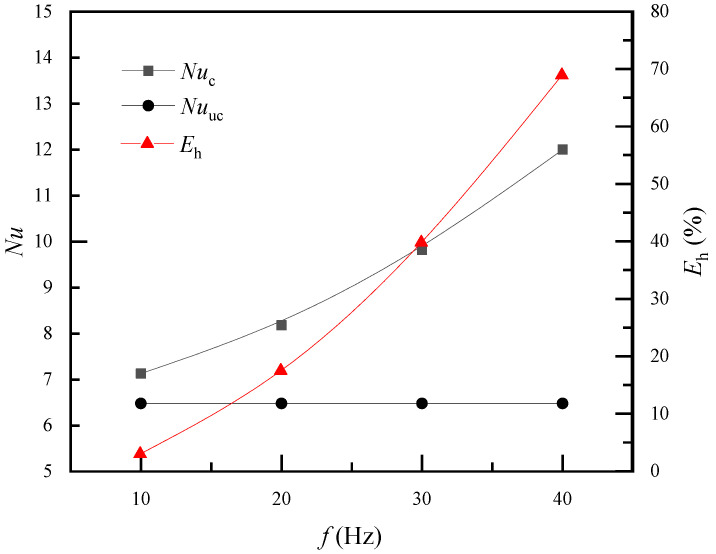
Vibrations of *Nu* with air pulsation frequency (*Re* = 165).

**Figure 9:**
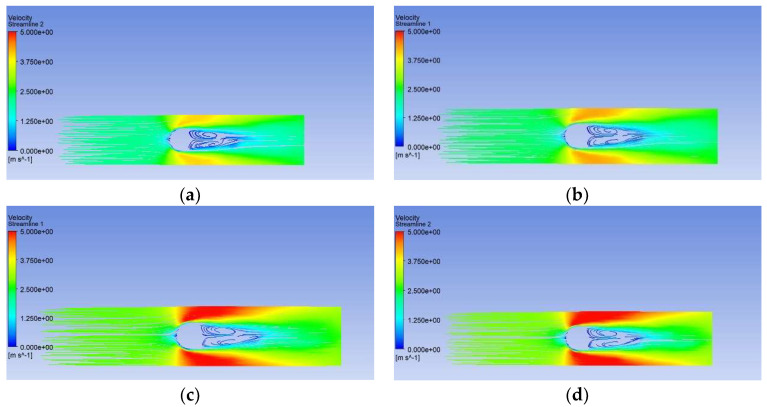
Streamlines for different incoming air pulsation frequencies (*Re* = 165). (**a**) 10 Hz. (**b**) 20 Hz. (**c**) 30 Hz. (**d**) 40 Hz.

Figure 8 shows the relationship between *Nu* and pulsating frequency. *Nu*_uc_ is a constant of 6.48 when the fiber is treated as a non-vibrating tube. When the fiber vibrates, *Nu*_c_ increases from 7.13 to 12.01 with increasing pulsation frequency. Convective heat transfer is usually enhanced by strengthened momentum transfer; therefore, the velocity field was analyzed. Hollow fibers will mainly vibrate toward the airflow direction, i.e., the *x* direction. Figure 9 depicts the streamlines for different pulsation frequencies at the times when the fiber returned to its original position. The contour plot is colored by air velocity magnitude. The velocity magnitude in the gap between the fiber and wall clearly increased with pulsating frequency. This means that with frequent vibration, the local maximum velocity increased and the high-velocity zone was enlarged. Velocity increments will reduce the thickness of the flow and thermal boundary layer. This may be the reason why the heat transfer coefficient increased with pulsating frequency.

### 4.5. Mass Transfer Performance Caused by Flow-Induced Fiber Vibration

The influences of air pulsating amplitude and frequency on mass transfer were also studied. Table 7 shows the effect of air pulsating amplitude on *Sh* number and *E*_m_. The *Sh* number changed little between vibrating and non-vibrating fiber conditions when A was 0.5 m/s. As the pulsating amplitude rose to 2 m/s, the *Sh* number under fiber vibration conditions was almost two times greater than the non-vibrating condition and the mass transfer enhancement index was 86.7%. The influence of air pulsation frequency on *Sh* number and *E*_m_ was consistent with the amplitude, and the calculated data are given in Table 8. As can be deduced, both increasing amplitude and frequency of the pulsating fluid can effectively enhance mass transfer flow across the hollow fiber membrane.

## 5. Conclusions

The flow-induced vibration of a hollow fiber membrane during humidification/dehumidification has been investigated using the FSI approach. Incoming air was assumed to exhibit pulsating flow with a sinusoidal waveform. The influence of the pulsating amplitude and frequency on the heat or mass transfer characteristics of the hollow fiber membrane was analyzed.

The fiber deformation was tested using a laser vibrometer. The maximum deformation of fiber in the airflow direction was about 5–10 times larger than that perpendicular to the flow direction. The *Nu* number under fiber vibration conditions increased by 14–87% compared with non-vibration fiber conditions, with a pulsating amplitude of 0.5–2 m/s. Flow-induced fiber vibration could raise the heat transfer enhancement index from 13.8% to 80%. The pulsation frequency could also enhance the heat transfer of hollow fiber membranes due to the continuously weakened thermal boundary layer.

The *Sh* number and *E*_m_ are also affected by flow-induced fiber vibration. At low amplitude or frequency, there is little difference in the *Sh* number under vibration and non-vibration conditions. As pulsating amplitude or frequency increased, the *Sh* number under fiber vibration conditions was almost two times greater than that under non-vibration conditions.

The purpose of this research was to investigate the effect of flow-induced vibration on the mechanical and heat and mass transfer performance of hollow fiber membranes in the humidification/dehumidification process. In this work, we tested the deformation of a single fiber in the experiment to verify the accuracy of the numerical model. Thus, the verified two-way FSI approach can be used to calculate flow-induced vibration in fiber bundles in future. The subject in flow-induced fiber vibration can be extended to a hollow fiber bundle rather than a single fiber. Choosing the characteristics of the hollow fiber membrane (length, diameter, Young modulus, Poisson’s ratio, etc.) that allows for a compromise between the heat mass transfer performance and the lifetime of the fiber under flow-induced fiber vibration is our future aim in this field of investigation. This will provide guidance for synthetic design of the membrane and the processing of the membrane module.

## Figures and Tables

**Table 1:** Dimensions of the computational domain.

Parameter	Symbol	Value	Unit
Fiber outer diameter	*d_o_*	1.7	mm
Fiber inner diameter	*d_i_*	1.5	mm
Fiber length	*l*	100	mm
Length of the airflow domain	*l*_a_	100	mm
Width of the airflow domain	*W*_a_	20	mm
Height of the airflow domain	*H*_a_	4	mm

**Table 2:** Amplitude and frequency of the pulsating flow in the simulation.

Serial Number	A (m/s)	B (Hz)	C (m/s)
1	0.5	20	1
2	1	20	1.5
3	1.5	20	2
4	2	20	2.5
5	1	10	1.5
6	1	20	1.5
7	1	30	1.5
8	1	40	1.5

**Table 3:** Parameter setups and fiber material properties in simulation.

Parameter	Symbol	Value	Unit
Density of air	*ρ*_a_	1.23	kg/m^3^
Viscosity of air	*μ*_a_	1.89 × 10^−5^	kg/m·s^−1^
Inlet air temperature	*T*_a_	35	°C
Heat conductivity of air	*k*_a_	0.024	W/m·K^−1^
Density of solution	*ρ*_b_	1218	kg/m^3^
Viscosity of solution	*μ*_b_	1.01 × 10^−3^	kg/m·s^−1^
Inlet solution temperature	*T*_b_	20	°C
Heat conductivity of the solution	*k*_b_	0.6	W/m·K^−1^
Young modulus of the fiber	*E*	280	MPa
The Poisson ratio of the fiber	*κ*	0.42	/

**Table 4:** Mesh independence check.

Grid Number	*d*_max_ (mm)	*T*_o_ (K)	*ω*_a_
257,165	0.63421	306.831	2.271%
281,738	0.62358	306.772	2.246%
293,025	0.61211	306.751	2.227%
333,445	0.61207	306.749	2.222%
393,558	0.61202	306.748	2.220%

**Table 5:** Experimental verification of maximum fiber deformation in *x* and *y* directions.

*Re*	Serial Number	*D_x_* (mm)			*D_y_* (mm)		
		Experimental	Numerical	Deviation	Experimental	Numerical	Deviation
110	1	0.138	0.140	−1.45%	0.023	0.024	−4.35%
	2	0.135		−3.70%	0.025		4.00%
	3	0.137		−2.19%	0.025		4.00%
	4	0.138		−1.45%	0.026		7.69%
	5	0.143		2.10%	0.024		0.00%
165	1	0.301	0.299	0.66%	0.049	0.049	0.00%
	2	0.292		−2.40%	0.052		5.77%
	3	0.289		−3.46%	0.047		−4.26%
	4	0.312		4.17%	0.05		2.00%
	5	0.310		3.55%	0.046		−6.52%
220	1	0.624	0.612	1.92%	0.056	0.054	3.57%
	2	0.596		−2.68%	0.054		0.00%
	3	0.613		0.16%	0.053		−1.89%
	4	0.605		−1.16%	0.052		−3.85%
	5	0.618		0.97%	0.055		1.82%
275	1	1.037	1.036	0.10%	0.105	0.103	1.90%
	2	1.015		−2.07%	0.102		−0.98%
	3	1.021		−1.47%	0.107		3.74%
	4	1.019		−1.67%	0.105		1.90%
	5	1.040		0.38%	0.101		−1.98%

**Table 6:** Maximum and minimum stress on the fiber for various *Re*.

*Re*	*τ*_min_ (MPa)	*τ*_max_ (MPa)	*τ*_mean_ (MPa)
110	1.73 × 10^−5^	0.0435	0.0097 MPa
165	2.03 × 10^−4^	0.2453	0.0540 MPa
220	7.61 × 10^−4^	0.6506	0.1372 MPa
275	2.14 × 10^−3^	1.0488	0.2138 MPa

**Table 7:** Effect of air pulsating amplitude on *Sh* number and *E*_m_.

*Re*	*A* (m/s)	*B* (Hz)	*C* (m/s)	*Sh*_uc_	*Sh*_c_	*E*_m_
110	0.5	20	1	3.46	3.96	12.0%
165	1	20	1.5	5.75	7.31	28.0%
220	1.5	20	2	8.24	12.8	55.2%
275	2	20	2.5	10.8	20.3	86.7%

**Table 8:** Effect of air pulsating frequency on *Sh* number and *E*_m_.

*Re*	*A* (m/s)	*B* (Hz)	*C* (m/s)	*Sh*_uc_	*Sh*_c_	*E*_m_
165	1	10	1.5	5.75	6.37	16.3%
165	1	20	1.5	5.75	7.31	28.2%
165	1	30	1.5	5.75	8.77	56.7%
165	1	40	1.5	5.75	10.7	96.2%

## Data Availability

Not applicable.
